# Cervical Pott’s Disease Presenting As Quadriplegia in a Young Patient: A Case Report

**DOI:** 10.7759/cureus.46949

**Published:** 2023-10-13

**Authors:** Sana Rahim Khan, Afaq Ahmad, Muhammad Saqib, Muhammad Iftikhar

**Affiliations:** 1 Department of Medicine, Khyber Teaching Hospital, Peshawar, PAK; 2 Department of Clinical Research, Kahuta Research Laboratory (KRL) Hospital, Islamabad, PAK

**Keywords:** spine tuberculosis, spinal tuberculosis:, young patient, humans, quadriplegia, cervical abscess, pott’s disease

## Abstract

Cervical Pott's disease, a form of spinal tuberculosis (TB), is a significant concern in low- and middle-income countries (LMICs). Early detection and treatment are critical to preventing complications like vertebral collapse. Clinical and radiological features of cervical Pott's disease can resemble other spinal conditions, posing diagnostic challenges.

We present a case of a 28-year-old female who initially presented with quadriplegia, cervical instability, and high-grade fever. Through multidisciplinary collaboration and prompt intervention, the patient was diagnosed with cord compression syndrome secondary to a cervical abscess and probable cervical Pott's disease. Anti-tuberculosis treatment (ATT) and steroids were initiated, leading to significant improvement in symptoms and disease resolution.

In summary, this case underscores the diagnostic challenge of cervical Pott's disease and the importance of imaging in TB diagnosis in resource-limited settings. The positive treatment response emphasizes early intervention's significance.

## Introduction

*Mycobacterium tuberculosis *(*M. tb*) is the second leading cause of infectious disease-related death worldwide [1]. In 2018, it infected 10 million people and caused 1.5 million deaths [2]. While TB primarily affects the lungs, it can, in rare cases, spread to the bones and joints, particularly the spine, known as Pott's disease. Diagnosing Pott's disease is challenging due to its varied clinical presentations, resulting in a delay of four to six months from symptom onset to diagnosis. This delay worsens the prognosis, often leading to surgery and neurological deficits. Although an MRI can detect spinal TB's soft tissue changes, it is typically used after neurological symptoms appear and is often confused with non-tuberculous spinal infections. Consequently, many patients may experience permanent neurological impairment once deficits develop [1].

Quadriplegia in young patients is a challenging clinical scenario. It can be attributed to a myriad of etiologies, including infections, neoplasms, and degenerative conditions [3]. Here, we present a case of a young female patient who initially presented with quadriplegia and was treated accordingly.

## Case presentation

A 28-year-old female was engaged in normal household activities when she suddenly experienced severe, sharp right shoulder girdle pain. The pain radiated to the right cervical region and was accompanied by monoplegic symptoms. These symptoms were inclusive of the curling of the hands and stiffness in the feet, which were accompanied by weakness, spasticity, numbness, and even episodes of paralysis in the right upper limb. Remarkably, despite the severity of these symptoms, a strong and steady pulse was maintained by the patient. Additionally, she developed a high-grade fever along with rigors and chills. Upon initial presentation, she was empirically treated as a case of meningoencephalitis. However, her condition continued to deteriorate. The patient was completely quadriplegic with cervical instability, urinary retention, and constipation. She had a persistently high-grade fever.

On clinical examination, the patient initially presented with the singularly affected right upper limb exhibiting weakness and diminished muscle tone, consistent with monoplegia. As the condition evolved, the paralysis progressed to encompass the entire side of the body, manifesting as hemiplegia, with further muscle atrophy and sensory changes. Subsequently, both lower limbs were paralyzed, resulting in paraplegia, characterized by a loss of motor function, decreased reflexes, and sensory deficits in the lower extremities. Finally, the condition advanced to quadriplegia, where all four limbs were paralyzed, marked by a profound loss of muscle tone, absent reflexes, and sensory deficits at the levels of C3, C4, and C5, necessitating a comprehensive diagnostic evaluation and immediate medical attention to identify the underlying cause and provide appropriate intervention. Power was 2/5 in all four limbs, and tone was the movement of muscles against gravity only. The patient appeared in distress, with facial expressions indicative of pain. The patient had a blood pressure of 120/80 mmHg, a pulse rate of 82 beats per minute, a respiratory rate of 19 breaths per minute, an oxygen saturation of 98% on room air, and a temperature of 100°F on arrival at our hospital. During the neurological examination, the patient displayed clear orientation concerning time, place, and person. Their Glasgow Coma Scale score revealed a relatively high level of consciousness, with an E4 for eye-opening, V5 for verbal response, and M1 for motor response. Additionally, their Mini-Mental Status Examination score was 28 out of 30, excluding any signs of confusion, indicating intact cognitive function. During the examination, cranial nerves were observed to be normal. Sensory levels remained intact, except for the levels of C3, C4, and C5, where sensory deficits were noted. Further clinical examination of the patient revealed normal findings across various systems. The cardiac examination indicated a regular rhythm with no murmurs or additional sounds. The abdominal evaluation demonstrated a soft, non-tender abdomen with normal bowel sounds and no organomegaly. A musculoskeletal examination revealed no joint abnormalities or muscle weakness. The dermatological assessment showed clear skin without any rashes or lesions. Eye examinations displayed reactive pupils and intact extraocular movements. The ENT examination confirmed the absence of ear, nose, or throat abnormalities. Head and neck examinations found no masses or lymphadenopathy.

On further history, there was a significant history of contact with active TB in the husband as well as living in an area endemic to TB. Moreover, the patient had a poor socio-economic background.

The patient’s laboratory investigations revealed pertinent findings, including a white blood cell count of 36,000/mm³, with 84.7% neutrophils and 10.1% lymphocytes. Hemoglobin (Hgb) was within the normal range at 15.4 g/dL. The mean corpuscular volume (MCV) was 84.8 fL, well within the normal range. The platelet count was recorded at 206,000 cells/mL, falling within the expected range. Fasting glucose levels were elevated at 137 mg/dL, slightly above the normal range. Hemoglobin A1C was 4.9%. Creatinine and blood urea nitrogen (BUN) values were 0.89 mg/dL and 11 mg/dL, respectively, both within the normal range. Potassium and sodium levels were 3.79 and 138 mmol/L, respectively, while chloride was 102.3 mmol/L, all within their respective normal reference ranges. Furthermore, the erythrocyte sedimentation rate (ESR) was elevated at 55 mm/h, indicating increased inflammation. Serum tests indicated a total bilirubin level of 0.81 mg/dL, alanine transaminase (ALT) at 34 U/L, and alkaline phosphatase levels of 181 U/L, all within the normal range. Testing for hepatitis B, hepatitis C, and HIV using enzyme-linked immunoassay (ELISA) yielded negative results. Notably, C-reactive protein (CRP) levels were also increased (50 mg/L), further highlighting the presence of an ongoing inflammatory process.

**Figure 1:**
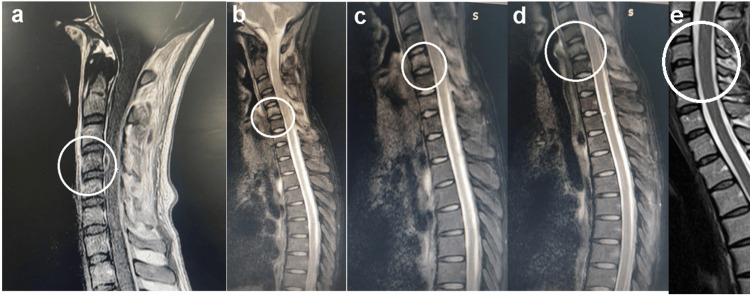
Cervical magnetic resonance imaging of the patient (a) T1 (recognizable by the dark cerebrospinal fluid (CSF)), (b) early T2, (c) T2-sequence showing vertebral enhancement, and (d) showing evidence of vertebral involvement, all revealing lesions at the level of C3, C4, and C5 vertebral levels. (e) shows the healing of the vertebral body after therapy. White circles in (a), (b), (c), and (d) show areas of infection, while those in (e) show healing. Patient identification is removed to ensure privacy.

The patient’s chest X-ray, echocardiography, electrocardiography, and ultrasound of the abdomen and pelvis were done, which revealed normal findings. The cervical spine MRI is shown in Figure 1.

## Discussion

The case under discussion revolves around the management of a 28-year-old individual displaying symptoms suggestive of cervical Pott's paraparesis. Initially, the patient presented with radiating pain and quadriplegia. It's important to note that in regions like Pakistan, spinal TB frequently manifests as an extrapulmonary condition, especially among younger individuals. Nonetheless, when patients present with progressive back pain and paraparesis, the suspicion often shifts towards metastatic lesions, even in areas with limited medical resources. Therefore, it is crucial, particularly in resource-constrained settings, to consider TB, or Pott's disease, as a potential diagnosis for patients of all age groups who exhibit radiating back pain and progressive paraparesis.

In the context of Pakistan, where TB is endemic, it is crucial for healthcare practitioners to maintain a heightened level of suspicion when encountering a patient with symptoms of radiating back pain and paraparesis [4-6]. In this particular scenario, numerous risk factors have been discerned, which include previous exposure to TB, a disadvantaged socio-economic background, and residing in an area characterized by a high TB prevalence. Furthermore, to bolster the clinical suspicion of spinal TB, it is imperative to perform spinal imaging. This not only serves to corroborate the diagnosis of Pott's disease but also augments the comprehensiveness of the clinical evaluation [4-6]. The patient's suffering is exacerbated by the fact that Pakistan is a resource-limited country. To undergo cervical spine imaging via MRI, the patient had to travel from their remote village to a distant city where such facilities were accessible. This journey, in addition to the medical expenses, places a significant financial strain on patients. Unfortunately, this aspect of disease management is often overlooked in more affluent nations where access to high-quality healthcare is much improved.

The recommended duration of anti-tuberculosis treatment (ATT) for individuals diagnosed with spinal TB can vary depending on the specific guidelines followed. According to the World Health Organization (WHO), the suggested minimum duration for ATT is at least nine months [7, 8], the American Thoracic Society recommends a range of six to 12 months, and the British Thoracic Society advises a six-month therapy [7, 8]. However, it's worth noting that certain specialists advocate for extended treatment durations, ranging from 12 to 24 months. This prolonged approach may continue until there is clear radiological or pathological evidence indicating regression of the disease [8-10]. Given the substantial disability and mortality associated with central nervous system (CNS) TB, it's strongly advised to consider using supplementary steroids, especially for individuals diagnosed with tuberculous arachnoiditis. In our specific case, we maintained the ATT for a duration of 12 months, ceasing oral steroid administration after eight weeks of therapy. Prolonging the ATT regimen is essential to ensure the complete eradication of *Mycobacterium tuberculosis*, thus mitigating the risk of developing multi-drug-resistant (MDR) TB. The patient received a comprehensive ATT regimen tailored to their weight and condition, which consisted of isoniazid at a daily dose of 300 mg, rifampicin at 450 mg, pyrazinamide at 1200 mg, and ethambutol at 1000 mg. Alongside this, intravenous dexamethasone was administered at a dosage of 24 mg per day for a period of three weeks. Dexamethasone, a potent corticosteroid, was prescribed to manage the inflammatory response associated with TB. The duration of hospitalization and the timeline of recovery will vary depending on the patient's specific condition and response to treatment, with regular monitoring and follow-up assessments being crucial to ensure progress toward recovery while minimizing potential side effects of the treatment. In our case, the patient spent two weeks in the hospital, followed by monthly hospital visits to monitor for successful treatment. Successful completion of the prescribed course of treatment is paramount to preventing drug-resistant strains of TB and ensuring a favorable outcome. Regarding Pott's disease, we can monitor the clearance of TB bacilli through a series of sequential spine MRIs. The absence of post-gadolinium enhancement in these scans signifies the absence of active disease, aiding in decisions to discontinue ATT. Surgery, in this context, is reserved for confirmed cases of Pott's disease at the microbiological or molecular level. These cases typically exhibit progressive acute neurological deficits, substantial spinal deformities surpassing 40 degrees of segmental kyphosis, notable anteroposterior or lateral translation, inadequate outcomes from medical treatment, or intense pain attributable to abscess formation or spinal instability [8, 11].

When physicians address CNS infections such as spinal TB, their primary guide for management should be clinical and radiological evidence. This approach is essential to avoid delays in treatment. It's crucial to have a grasp of the anatomical and pathophysiological factors contributing to a typical tuberculous lesion, as this knowledge aids in differentiating it from conditions that mimic spinal TB. Specifically, the presence of paradiscal lesions, the engagement of the anterior segment (body) of vertebrae, and the destruction of the intervertebral disc are strong indicators of Pott's spine [12].

In this case, key differential diagnoses included metastatic spinal lesions, pyogenic spinal infections, and degenerative diseases [13-16]. The diagnosis of Pott's disease was supported by the involvement of the anterior vertebral body and intervertebral discs [8]. In contrast to metastatic carcinomas, which tend to impact the posterior vertebral elements, pyogenic spinal infections frequently present with acute toxic symptoms. However, in this particular case, the symptoms manifested over a two-week period without evident toxic features. Furthermore, it was less likely that degenerative vertebral diseases were at play, as they typically affect distinct regions and lack specific abnormalities [17].

To summarize, this case serves as an example of Pott's paraplegia, likely stemming from spinal TB. Swift administration of anti-TB medications and steroids resulted in notable improvements both clinically and radiologically. This underscores the significance of utilizing imaging for TB diagnosis, especially in situations where microbiological tests are unavailable. It also highlights the positive outcomes associated with early intervention.

This case emphasizes the necessity of promptly considering uncommon causes, even in young patients experiencing quadriplegia. It underscores the importance of employing contrast-enhanced radiological examinations when renal function permits and highlights the value of a multidisciplinary approach in achieving precise diagnoses and timely interventions.

## Conclusions

A timely and accurate diagnosis is crucial for young patients presenting with quadriplegia. This case demonstrates the successful management of a rare case of impending cord compression secondary to cervical Pott's disease through a rapid clinical approach, highlighting the significance of prompt intervention to prevent permanent paralysis.
